# Potential Toxicity of Iron Oxide Magnetic Nanoparticles: A Review

**DOI:** 10.3390/molecules25143159

**Published:** 2020-07-10

**Authors:** Nemi Malhotra, Jiann-Shing Lee, Rhenz Alfred D. Liman, Johnsy Margotte S. Ruallo, Oliver B. Villaflores, Tzong-Rong Ger, Chung-Der Hsiao

**Affiliations:** 1 Department of Biomedical Engineering, Chung Yuan Christian University, Chung-Li 32023, Taiwan; nemi.malhotra@gmail.com; 2 Department of Applied Physics, National Pingtung University, Pingtung 90007, Taiwan; jslee@mail.nptu.edu.tw; 3 The Graduate School, University of Santo Tomas, Manila 1015, Philippines; rhenzalfredliman@gmail.com (R.A.D.L.); ruallo.jm@gmail.com (J.M.S.R.); 4 Department of Biochemistry, Faculty of Pharmacy and Research Center for Natural and Applied Sciences, University of Santo Tomas, Manila 1015, Philippines; obvillaflores@ust.edu.ph; 5 Department of Bioscience Technology, Chung Yuan Christian University, Chung-Li 32023, Taiwan; 6 Department of Chemistry, Chung Yuan Christian University, Chung-Li 32023, Taiwan; 7 Center for Nanotechnology, Chung Yuan Christian University, Chung-Li 32023, Taiwan

**Keywords:** magnetic nanoparticle, toxicity, aquatic organism, rodent, cell

## Abstract

The noteworthy intensification in the development of nanotechnology has led to the development of various types of nanoparticles. The diverse applications of these nanoparticles make them desirable candidate for areas such as drug delivery, coasmetics, medicine, electronics, and contrast agents for magnetic resonance imaging (MRI) and so on. Iron oxide magnetic nanoparticles are a branch of nanoparticles which is specifically being considered as a contrast agent for MRI as well as targeted drug delivery vehicles, angiogenic therapy and chemotherapy as small size gives them advantage to travel intravascular or intracavity actively for drug delivery. Besides the mentioned advantages, the toxicity of the iron oxide magnetic nanoparticles is still less explored. For in vivo applications magnetic nanoparticles should be nontoxic and compatible with the body fluids. These particles tend to degrade in the body hence there is a need to understand the toxicity of the particles as whole and degraded products interacting within the body. Some nanoparticles have demonstrated toxic effects such inflammation, ulceration, and decreases in growth rate, decline in viability and triggering of neurobehavioral alterations in plants and cell lines as well as in animal models. The cause of nanoparticles’ toxicity is attributed to their specific characteristics of great surface to volume ratio, chemical composition, size, and dosage, retention in body, immunogenicity, organ specific toxicity, breakdown and elimination from the body. In the current review paper, we aim to sum up the current knowledge on the toxic effects of different magnetic nanoparticles on cell lines, marine organisms and rodents. We believe that the comprehensive data can provide significant study parameters and recent developments in the field. Thereafter, collecting profound knowledge on the background of the subject matter, will contribute to drive research in this field in a new sustainable direction.

## 1. Introduction

The field of nanotechnology has attracted the attention of scientific community worldwide. The advancement in the field in form of different nanoscale chemical structures such as magnetic nanoparticles, quantum dots, fullerenes and metallic nanoparticles (less than 100 nm in dimension) have gained a lot of attention in the medical, electronic and material sciences. It was December of 1959, when Nobel Laureate Richard Feynman introduced the concept of nanotechnology in a lecture at the California Institute of Technology [1]. In the following years a lot has been done in the field to explore many aspects of nanomaterials and the engineering of these small nano-surfaces, in terms of their chemical composition, surface chemistries, binding ligands, antibodies for specific activities and reduction of toxicity [2]. Some nanomedicines have been approved by the U.S. Food and Drug Administration (FDA) and many others are in their final development stages [3]. Magnetic resonance imaging (MRI) contrast agents as such Sinerem^®^ designed using superparamagnetic iron oxides (SPIO) for lymph node imaging after promising initial data, failed to demonstrate consistent and statistical significant benefit for sensitivity in the pivot study [4,5,6], whereas, ferumoxytol, an ultra-small iron oxide nanoparticle approved to treat iron deficiency (anemia) in adults with chronic kidney disease resulted in no anaphylaxis. Also no life threatening adverse events were recorded [7] and hence ferumoxytol is available for safe usage within FDA approved guidelines [8].

Magnetic nanoparticles (MNPs) are a branch of nanoparticles made up of pure metals, metal alloys and metal oxides [9,10,11]. Iron oxide MNPs are the most preferable nanomaterials in medical sciences, due to their features of minimal toxicity, and excellent physiochemical properties such as superparamagnetism, stability in aqueous solutions and biocompatibility [9,11,12,13]. The stability of the magnetic response of iron oxide is attributed to its low sensitivity to oxidation [14]. Also, size control, prevention of aggregation by coating, specific interaction and dispersion, penetration of cell and tissue barriers all provide iron oxide MNPs an edge over other metal nanoparticles. Iron oxide MNPs provide a platform for theranostics, where they can be exploited for their contrast agent qualities in magnetic resonance imaging diagnostics, as well as for therapeutics in the form of drug delivery platforms inside the body, bio-catalysis, protein purification and magnetic cell separation [9,15,16].

A lot of methods have been proposed for the synthesis of magnetic nanoparticles, such as co- precipitation, reverse micelles synthesis, microemulsions, thermal decomposition or reduction, hydrothermal synthesis and laser pyrolysis [9,14,17,18]. After the synthesis of pure MNPs, in order to make them uniformly disperse in solvents and be more biocompatible, different polymers and surfactants, e.g., polyethylene glycol (PEG) [19,20], dextran [21,22], polyvinyl alcohol (PVA) [23,24] starch, chitosan, oleic acid and sodium dodecyl sulphate (SDS) coatings have been applied [12,15,25,26], respectively. Many studies have documented the features of nanoparticles such as large surface to volume ratio, rendering them more biologically reactive [27,28,29], because a large surface area in turn provides a huge number of active sites for interaction, which may in turn yield unfavorable responses [29,30,31].

**Figure 1:**
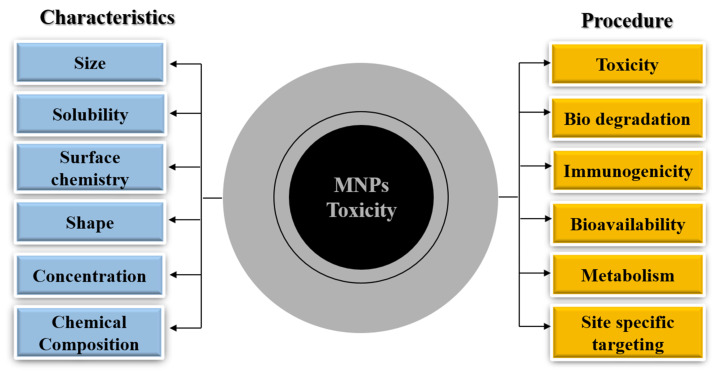
Characteristic properties of magnetic nanoparticles (MNPs). Physical properties like particle size, solubility, surface chemistry, shape, concentration, and chemical composition, determined at a broad scale to analyze subsequent toxicity caused by them in cell lines (normal/contaminated) and model organisms are summarized in the left panel (highlighted by blue color). The procedures to observe the underlying mechanism of toxicity, immunogenicity, metabolism, bioavailability and biodegradation under different parameters for designing MNPs more specifically for biomedical applications safely are summarized in the right panel (highlighted in orange color). See the Table 1 summary for detailed literature citations.

It is therefore important to understand the toxicity of MNPs, as it depends on numerous factors, e.g., size, shape, structure, surface modification, concentration, dosage, biodistribution, bio- availability, solubility, immunogenicity and pharmacokinetics [32]. The large surface to volume ratio may favor interactions within various bodily components, if inhaled, absorbed or swallowed, also, the ability to cross cell barriers and resistance to biodegradation increase the toxic potential of these nano-entities [14,32]. Toxicity studies should consider the underlying mechanism of acute as well as chronic toxicity, degradation of particulate products, any subsequent stimulation of reaction in defensive responses, inflammatory responses, metabolism and long term toxicity, and elimination in cellular as well as animal models [32] (concepts are summarized in Figure 1).

In the current review paper, we aim to discuss the toxicity parameters of iron oxide MNPs which have been studied to date in cell lines, aquatic organisms and rodent animal models and have provided some concrete relevant results. We hope this review will allow researchers in this field to contemplate different parameters of toxicity analysis and define future directions for useful application in animal models, which can lead to much help in humans at later stages.

## 2. Formulation of Magnetic Nanoparticles

Comprehensive arrays of magnetic materials are available. Iron oxide-based materials such as magnetite and maghemite are considered safe and are also currently in clinical use as MRI contrast agents [33]. Iron-based metal oxides exhibit strong magnetic properties and are usually used for producing MNPs. Iron alone is susceptible to rusting and corrosion, hence cannot be used as a core material for the synthesis of MNPs without protective, strong and inert coatings. Magnetite (Fe_3_O_4_) and maghemite (γ-Fe_2_O_3_) are considered the most suitable materials for synthesis of MNPs. They differ in their iron oxidation states, which changes their physiochemical properties [34]. Maghemite is also the most preferred material for the MNP cores because it is least likely to cause any health hazards and as iron (III) ions are already found in the human body, any percolation of the metal will not cause any substantial side-effects [35]. To maintain the stability of nanoparticles in aqueous medium is a foremost challenge [36]. The core of MNPs possess a high surface to volume ratio, and thus preventing the interaction of the MNP surface within the body during in vivo applications is necessary. For this purpose, coating MNPs is done to shield them from the surrounding environment with the help of natural (carbohydrates, proteins), synthetic polymers (polyethylene glycol (PEG), polyvinyl alcohol (PVA), polyvinyl pyrrolidone (PVP), poly lactic-co-glycolic acid (PLGA) and noble materials (gold, silver) [34,37] to provide them better stability, sustainability and mechanical strength [36,38].

### Desirable Properties of Magnetic Nanoparticles

Monodispersity (similar shape and size), superparamagnetism, biocompatibility, surface chemistry are some of the key desired properties of MNPs [32]. The synthesis of MNPs with these particular qualities has been an active area of research for quite some time now [39]. The synthesis of MNPs with controlled shape and size has been demonstrated in different studies [40,41,42,43,44]. MNPs can be synthesized using various defined techniques, namely, hydrothermal synthesis, sol-gel reaction methods, microemulsion methods, electrospray synthesis [45], chemical co-precipitation, thermal decomposition, solvothermal processes, sonochemical synthesis, microwave assisted synthesis, chemical vapor deposition, carbon arc, combustion, laser pyrolysis, [46,47] spray pyrolysis, precipitation from solution, polyol method, green synthesis [48], bacterial and microorganism synthesis (including magnetotactic bacteria and iron reducing bacteria) [49,50] and electrochemical synthesis [48]. Superparamagnetism is a characteristic of MNPs which depends upon the presence or absence of an external magnetic field. This magnetism phenomenon occurs in small ferromagnetic and ferrimagnetic nanoparticles; in these nanoparticles the magnetization flips randomly under the influence of temperature. The time between two flips is called the Neel relaxation time. In the absence of an external magnetic field if the time used to measure the magnetization of nanoparticles is more than the Neel relaxation time, the average magnetization appears to be zero, and the nanoparticles are said to be in a superparamagnetic state [51]. According to the observations of Hofmann-Amtenbrink et al., ferromagnetic materials remain magnetised and exhibit strong attraction to magnetic fields, even after the removal of the external magnetic field [52]. Superparamagnetism is important feature to avoid agglomeration and direct MNPs to site-specific locations inside the body. Superparamagnetism arises from the core magnetic material in the MNPs, which is more importantly based on the size of particle. Biocompatibility and surface functionalization are other requirements for MNPs to be stable during in vivo applications, which can be achieved by coating the core particle with different polymers [53,54].

The small constituent units of monomers undergo polymerization to form polymers. On the basis of polymer chain structure polymers can be divided into linear, branched, cross-linked and networks [55]. Polymers have unique properties, which are based on the binding of each molecule individually inside them. The process of chemical bond formation, under defined temperature and pressure conditions, make the single units of polymers bind in linear as well as branched manners, resulting in bending as well as stretching properties. Polymers can be categorized as natural as well as synthetic polymers, and biodegradable or not [56]. Natural polymers tend to occur naturally in nature and synthetic polymers can be synthesized in the laboratory. Examples of some natural and synthetically formulated polymers are starch, gelatin, dextran, chitosan, cellulose and polyethylene glycol (PEG), polyvinyl alcohol (PVA), polyvinylpyrrolidone (PVP), poly (acrylic acid), poly(*N*-vinylpyrrolidine), polyanhydrides, polycaprolactone, poly(*N*-isopropylacrylamide) (NIPAAm), polypyrrole (PPy), poly(lactic-co-glycolic acid) (PLGA), polylactic acid (PLA), etc. [34,57,58,59,60,61,62]. The polymers PLGA and PLA are FDA approved based on the simplicity of their particle formulation and non-toxic biodegradation products, which is why they are the most preferred in drug delivery particle formulations [59,63,64,65]. These polymers are used to coat the unstable reactive surface area of MNPs for stabilize them for in vivo functions like drug delivery or gene delivery by adsorbing proteins or loading drugs [34]. The superparamagnetic core of these fabricated molecules helps these molecules accumulate at a desired site with the help of an external applied magnetic field and unload the drug molecule at specific sites [34,66,67,68,69,70]. Surface coating with PEG has been shown to reduce the interaction of MNPs with plasma proteins, decreasing the chances of internalization and clearance by macrophages [62,71]. Noble metals such as gold (Au) and silver (Ag) are another class of materials which are used to coat MNPs. Gold nanomaterials which are assembled with magnetic iron oxides to provide gold-iron oxide hybrid structure molecules, offer benefits like good drug delivery, spin dynamics and plasmonic applications as gold nanomaterials possess various properties like catalysis, photothermal properties and plasmonic resonance [72,73]. Similarly, silver-iron oxide hybrid nanomaterials when generated which demonstrated surface enhanced plasmonic resonance, antibacterial and catalytic properties [72,74].

## 3. MNP Metabolism and Toxicity

### 3.1. MNP Biodistribution and Metabolism

The surface chemistry and course of delivery of MNPs each influence their biodistribution patterns and circulation time in the body [62]. MNPs which are more than 200 nm in size are known to be captured by the spleen via mechanical filtration, whereas MNPs of less than 10 nm can be removed through renal clearance, hence 10–100 nm is defined as an optimal range for administration for specific applications [17]. The biodistribution patterns of these particles has been defined as 80–90% in liver, 5–8% in the spleen and 1–2% in bone marrow [75]. The interaction and biodistribution of MNPs’ surface and the mechanism of internalization inside cells and organs, metabolism and possible toxicity all depend on any surface modifications [17,76,77,78]. In previous works Soenen et al. [33,34] already conducted very systematic toxicity assessments of iron oxide nanoparticles of different sizes and surface functionalization at a cellular level. In this review, we wish to further review the literature on the potential toxicity caused by exposure to iron oxide nanoparticles of different sizes and surface functionalization at the cellular, tissue and whole organism levels (a side-by-side comparison is provided in Table 1).

Fluorescent magnetic nanoparticles (FMNPs), when inhaled by ICR mice in low concentration 4.89 × 10^5^ ± 2.37 × 10^4^/cm^3^ and high concentration 9.34 × 10^5^ ± 5.11 × 10^4^/cm^3^ for four weeks in a nose-only exposure system, were found majorly in the liver, testis, spleen lung and brain indicating the crossing of the blood brain barrier (BBB) and blood testis barrier (BTB) [104]. In another study when suspensions of MNPs were injected via the tail vein (10 mg Fe/mL) the biodistribution of iron and its clearance were demonstrated to be dependent on complex events and dynamics of iron concentration in different tissues with changes over time. Thus, injected iron did not caused long term changes in liver enzyme levels or oxidative stress [105]. When polyethylene glycol-coated magnetic nanoparticles (PEG-MNPs) were injected in 1 mm diameter burr holes drilled in male Fisher 344 rats’ skulls for intracerebral brain tumors the results suggested that liver biodistribution was less important in comparison to other studied MNPs and instead spleen biodistribution and sustained accumulation of PEG-MNPs raised toxicity concerns. The research group hence suggested a long time study to assess the toxicity risks [106]. In a further study, intraperitoneal injection with 3-aminopropyl-triethoxysilane-coated magnetic nanoparticles (APTS-MNPs) into ICR mice showed the biodistribution of iron in various body tissues to change with time (analyzed at 1, 2, 3 and 4 weeks), where a greater fraction of iron was localized in the liver and spleen, not causing continuing changes in liver and kidney function with time and hence it was concluded they were safe for in vivo application usage, although the concluding remarks of the study suggested the work to be inadequate and suggested more investigations be done at a molecular level [107]. The binding of MNPs to exogenous structures may cause different responses and influence their uptake and metabolism depending on the organ or cell type [108]. A study demonstrated that CD301b molecules marked different set of MNPs in various organs which suggested involvement in systemic metabolism including the adipose tissue, skeletal muscle, colon, and pancreas [109].

### 3.2. MNP Toxicity

**Figure 2:**
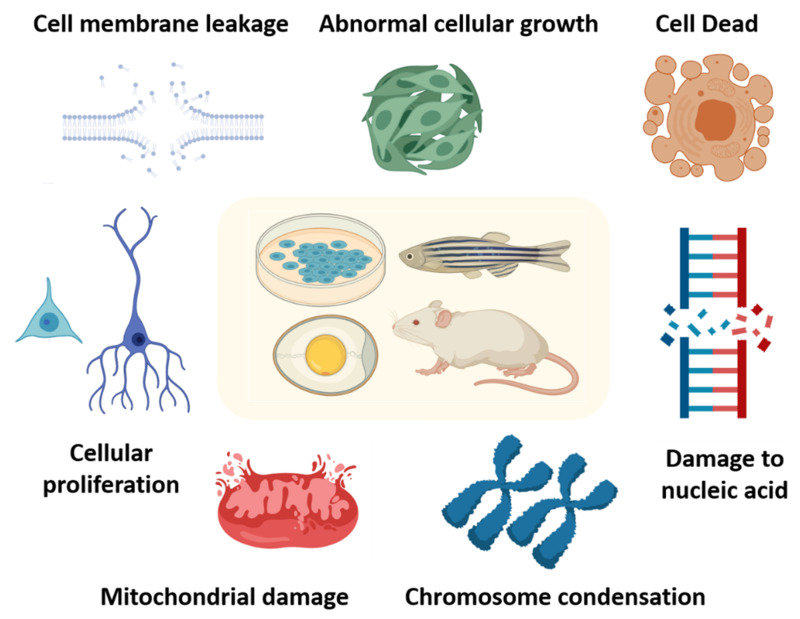
Potential toxicity effects of magnetic nanoparticles (MNPs) collected from in vitro and in vivo experiments. The common strategies for risk assessment for MNPs, including cell culture, zebrafish, chicken and rodents are summarized in the central yellow panel. The potential toxicity effects of MNPs have been categorized by mechanism, such as inhibition of cellular proliferation, temporary/absolute cell cycle cessation, DNA genotoxicity, damage of cellular components and aggregation to blocking of cell membranes on a cellular level. Some studies have reported MNPs’ toxicity effects on a whole organism level and elaborated the interactions of MNPs inside the body (see the summary in Table 1 for detail literature citations).

When considering the usage of MNPs for future healthcare applications, toxicity is the major area of concern, as not much exploration has been done in this area [108]. Toxicity studies usually define the adverse effects in terms of physical, chemical and biological agents in tested animals and the environment [110]. Also, the toxic nature of these particles can result in diminished therapeutic efficiency [111] and migration and accumulation of MNPs in organs which can in turn activate inflammatory or immune responses [112]. If these MNPs potentially enter inside the cell, the toxicity can affect the nuclear activities or cause leakage or blockage of cell membranes which can lead to adverse cell proliferation, viability and metabolic activity results [113]. Therefore, studies of toxicity for all manufactured MNPs are required in all their dimensions (concepts are summarized in Figure 2).

## 4. MNP Toxicity In Vitro and In Vivo

### 4.1. MNP Toxicity In Vitro

The safety valuation of MNPs on cell lines (in vitro) is an easy, simple and inexpensive method as experiment can be controlled consistently [114,115]. MNPs toxicity of iron oxide nanoparticles (IONPs) have been linked to criteria like dose-dependency, time, surface modification [116], concentration [117,118,119], size [120,121] and shape [122] parameters. The higher the number of nanoparticles, the higher risk they possess for toxic effects [123]. These assays need to be examined very carefully, because the interaction of nanoparticles can interfere with the cellular components and their basic activities causing inappropriate statistics [124] for, e.g., if nanoparticle increases the generation of reactive oxygen species (ROS) that can in turn effect the activity of mitochondrial enzymes disturbing the results of 3-(4,5-dimethylthiazol-2-yl)-2,5-diphenyl tetrazolium bromide MTT assay [123,125]. On similar lines, it was demonstrated that classical dye based assessment MTT and neutral red (NR) assays generated misleading results because of the interactions between some nanoparticles and dyes [126]. Some of the most used techniques in in vitro assays for analyzing cell viability, proliferations, differentiation are optical microscopy, electron microscopy, atomic force microscopy on the basis of image observations of nanoparticles internalization in the cells at very small scale range of nanometers, which can be further used to analyze data through, analytical software’s such as ImageJ [127]. Also, gene expression analysis [128,129], proteomics, and metabolomics are new avenues facilitating to study the underlying mechanism of toxicity. Studying the cell on all these parameters is valuable for initial biocompatibility, MNPs-cell membrane interaction and aggregation testing and base indication of any physiological effect.

Spherical shaped Fe_3_O_4_ MNPs were characterized in a size range of 72.6 ± 0.6 nm. When tested in vitro to study the eryptosis indices as an analytical measure to see the effect on red blood cells (RBCs) in cellular membrane microstructure and cellular function, 25 µg/mL Fe_3_O_4_-MNPs caused noteworthy impairment to erythrocytes. Later when tested with female CD^®^ IGS Rats at 8 weeks of age Fe_3_O_4_ MNPs at a concentration of 12 mg/kg led to apoptosis of circulating erythrocytes in vivo. Overall, notable changes of Fe_3_O_4_ MNPs were able to cause changes in the mechanical properties of erythrocytes, with pathological changes in cell membranes, abnormal cytosolic calcium levels, and oxidative stress also triggering programmed cell death both in vitro and in vivo [79]. In other study model, uncoated Fe_3_O_4_, Na-oleate-coated Fe_3_O_4_ (OC-Fe_3_O_4_), fluorescent rhodamine-labeled silica 25 nm and 50 nm (Fl-25 SiO_2_, Fl-50 SiO_2_), respectively, were used to evaluate toxicity parameters in BeWo b30 placental cell barrier model, through means of lactate dehydrogenase (LDH) leakage in the medium, at a concentration range of 75, 15, 3, 0.6 and 0.12 µg/cm^2^ for 4, 24 and 48 h. The results indicated that cytotoxicity of iron oxide was apparent at low doses after a short time of exposure in comparison to silica particles [82]. Similarly, when uncoated MNPs and *n*-octyltriethoxysilane- coated MNPs were tested on PC12 (rat pheochromocytoma) and ReNcell VM (human neural stem cells), at a concentration of 0–64 µL for 24 h, the results indicated decreased cytotoxicity effects from coated MNPs. Uncoated MNPs had decreased association in PC12 cells but not in RVM cells. This study further indicated significant differences in cytotoxicity profiles in consideration of cell type or the presence of serum matter for nanoparticle toxicology studies [102]. Besides, about 20–60 nm uncoated MNPs (1 ng/mL) were utilized to treat NRK-52E cell lines (rat, kidney). Via a comparative proteomics technique [130], the results showed that the rat-related proteins were enhanced; meanwhile, glutathione-related proteins and chaperone proteins were enhanced to protect NRK-52E cell lines, avoiding apoptosis. These previous studies show that MNP treatment caused a reduction in reactive oxygen species (ROS), apoptosis, and protective proteins were also enhanced to resist apoptosis [131].

In the next study, four different types MNPs, namely uncoated-magnetite MNPs (cationic), starch-magnetite MNPs, dextran-magnetite MNPs, uncoated-maghemite MNPs (anionic) were used for toxicity analysis on rat pheochromocytoma PC12 cells at concentrations from 0.01 to 0.5 mg/mL for 1, 2, 3, 24, 48 and 72 h. When an XTT test was performed for the analysis of cytotoxicity, the results suggested that different types of MNPs interact differently with cells. Uncoated magnetite particles did not enter the cells, just sitting on the outer surface. They showed no cytotoxic effect up to 0.1 mg/mL, whereas 51% cells remained viable at 0.25 mg/mL after 72 h. Starch magnetite particles formed non-homogeneous aggregates and a slight reduction in cell viability to 70% at 0.1 mg/mL was observed after 72 h. The trend remained the same for dextran-coated particles with a reduction in cell viability at a concentration of 0.25 mg/mL. Only the uncoated maghemite iron oxide nanoparticles demonstrated maximum interaction and penetration in cells with no cytotoxic effect at any tested concentration. The four groups examined here indicated the time of incubation and concentration of MNPs as toxicity-dependent criteria [90]. Maghemite anionic nanoparticles when tested on human prostatic tumor cells (PC3), where they were internalized by the cells and concentrated within the vesicles. On exposure to an alternating current (AC) field, these nanoparticles generate heat from inside the cells. Results showed 44% of cells were killed within one-hour of air conditioning (AC) exposure at 700 KHz-31 mT. These findings pave the way for effective cell killing facilitated by intracellular magnetic hyperthermia. Magnetic labelling with anionic MNPs does not cause any detectable cytotoxicity [132]. In accordance to both the mentioned studies, maghemite MNPs are suggested as safe as they did not produce any significant toxicity, even over longer time periods.

In one interesting study, MNPs-mediated magneto-mechanical stimulation of human primary adipose-derived stem cells that were exposed to variable magnetic field (MF) was shown to influence their adipogenic and osteogenic differentiation. Adipose tissue-derived stem cells (ADSCs) loaded with biocompatible magnetite nanoparticles of 6.6 nm in size and with an average load of 21 picograms iron/cell were exposed to variable low intensity (0.5 mT) and higher intensity magnetic fields (14.7 and 21.6 mT). The type, duration, intensity and frequency of the MF differently affected differentiation. Short time (2 days) intermittent exposure to low intensity MF increases adipogenesis, while a longer duration (7 days) exposure promotes osteogenesis. MNPs uptaken by ADSCs promote a shift towards an osteoblastic lineage. ADSCs- MNPs under MF exposure could thus be used for enabling osteoblastic conversion during cell therapy for systemic osteoporosis. Therefore, further in vivo studies are needed to discover ways to treat osteoporosis [133].

Green and one-pot synthesis of bare and amino acid-coated MNPs was used for cytotoxicity studies on a HFF2 cell line at a concentration of 0.049, 0.073, 0.110, 0.165, 0.248 and 0.373 mg/mL for 72 h. Here no cytotoxicity was observed and MNPs were biocompatible with the cells [93]. The synthesized MNPs were coated with a triblock copolymer (PEO-polyurethane-PEO), with two different chain sizes of 5 and 15 KDa and tested on different cells, namely human umbilical vein endothelial cells (HUVECs), human retinal pigment epithelial (HRPE), C4-2 metastatic human prostate adenocarcinoma cells, and PC3 prostate cancer cells. The results of the study indicated that the cytotoxicity of short chain copolymers is much higher in comparison to long chain polymers, and that the cause of toxicity is the polymer coating and not the magnetite core which is a desirable aspect for maintaining an increased amount of magnetic component for large magnetic moments [134]. Dextran- and PVA-coated iron oxide MNPs when tested on brain-derived EC219 endothelial cells and murine N9 and N11 microglial cells did not cause cytotoxicity [135]. Ferumoxtran-10, an ultra-small dextran-coated superparamagnetic iron oxide particle, did not show any toxic effect for human monocyte-macrophage interactions at a concentration of 1 mg/mL for over 72 h [136]. After controlling the shape and size of nanoparticles coated with different concentrations of PVA and stirring a subsequent MTT assay specified that the biocompatibility of nanoparticles based on cell viabilities can be improved by increasing their r-ratio, irrespective of the stirring rate, which can lead to growth of particle hydrodynamic size and cause lower cell toxicity effects [122]. To enhance their surface functionalization and surface charge features MNPs were coated with dextran, aminodextran, heparin, and dimercaptosuccinic acid (DMSA). These nanoparticles were then incubated with HeLa cells. The cationic dextran-coated MNPs provided efficient HeLa cell labeling for longer periods, detected using optical microscopy and did not alter the cell viability. On the contrary, anionic DMSA- coated nanoparticles were taken up by cells less efficiently and neutral charged nanoparticles were not taken up by cells. The research group concluded that nanoparticles with different surface charges are internalized by the cancer cells differently; also cationic MNPs show excellent properties for in vivo biomedical applications, such as cell tracking by MRI. [116]. DMSA-coated MNPs when studied on NCTC 1469 non-parenchymal hepatocytes, had no significant effect on cell viability, oxidative stress, cell cycle or apoptosis at a concentration of 0.5 mg/mL [137]. Both the studies with DMSA- coated MNPs indicate that a similar coating can also generate different results in accordance to the treated cell line.

### 4.2. MNPs Toxicity In Vivo

The interactions of MNPs inside biological systems are dynamic and complex [138,139,140]. The size of MNPs plays a very crucial role once inside the body. MNPs of less than (<10 nm) are removed through renal extravasation, whereas large particles (>200 nm) are captured by the spleen [17], therefore, MNPs in range from ~10–100 nm are the most preferred size to be used inside the body. Altogether, size, shape, surface charge and stability are some of the factors which determine the overall interaction of MNPs inside the body of the organism. The study of pharmacokinetic aspects like absorption, distribution, metabolism and excretion in the system of an organism is crucial to design nanoparticles with specificity towards cells and tissues, their metabolism and clearance criteria to understand the potential toxicity. Inside the body the MNPs can be absorbed through interactions with cells and other biological components; from there they can be distributed into various organs, and further metabolized. The most important biosafety phenomenon, which needed to be studied is how these MNPs interact and interfere with physiological iron metabolism after the degradation of these nanoparticles inside the body, as excessive accumulation of intracellular iron can damage the cellular components of cells such as proteins and nucleic acids.

### 4.3. MNP Toxicity in Invertebrates 

To evaluate the different aspects of MNPs, the need to start from the very basic level of the ecological pyramid is important, as these small organisms also play significant role in the ecological balance and food chains. These small model organisms may very well provide the direction of the research on testing of some specific small set criteria. In a study of Fe_2_O_3_ magnetic nanoparticles and arsenate (As (V)) synergistic effects were observed in *Ceriodaphnia dubia* (water flea). When exposed to MNPs, the bioaccumulation of MNPs increased with the increase in concentration up to 20 mg/L within a time period of 6 h, but later a decrease in accumulation of MNPs resulted due to settling and aggregation of nanoparticles. Also, no toxicity and mortality was observed for nano-Fe_2_O_3_ alone under any tested concentration in the study, but after the addition of As (V) the toxicity was enhanced [141]. Similarly, when a toxicity evaluation was done comparing two types of MNPs (MNP and MNP integrated with zeolite) in mussels (*Mytilus galloprovincialis*) for 1, 3 and 7 days, both the MNPs induced changes in the animal physiology at a concentration range of 10 and 50 mg/L of iron oxide NPs and 50 and 100 mg/L of iron oxides incorporated with zeolite by causing oxidative stress in hemocytes of exposed mussels in comparison to control group. Also, increases in ROS, lipid peroxidation, DNA damage, ubiquitin conjugate, and protein carbonylation were observed on the 7th day with iron oxide incorporated zeolite NPs [142]. Next, in another study the size effects on adsorption of hematite NPs was studied in *E. coli* cells. In this particular study it was specified that adsorption of hematite (α-Fe_2_O_3_) NPs (76 and 96 nm) on *E. coli* cells reached equilibrium faster (30–40 min) in comparison to small NPs which took 60–90 min. The decreasing size order rate in mg Fe/L was 98 nm > 76 nm > 53 nm > 26 nm. The results obtained in this paper can be considered as a ground work for developing models to describe the thermodynamics and kinetic behavior of a range of NPs towards adsorption in microorganisms. This results obtained in this particular study used an amalgamation of Extended Derjaguin-Landau-Verwey-Overbeek (EDLVO) and Interfacial Forced Boundary layer (IFBL) theories to provide a quantitative method to define thne adsorption kinetics of hematite NPs on bacterial surfaces at the nanoscale [143].

As stated earlier, toxicity studies in small invertebrate models is important because they are major elements of maintaining balance between ecological chain and the surrounding environment. Along these lines, a study presented the effect of iron oxide MNPs deterioration of the mutual relation between the fungus *Arbuscular mycorrhizal* and plants, where Fe_3_O_4_ MNPs were proved to be toxic to fungi at high concentrations of 10.0 mg/kg, with a destructive impact on the mutual interaction between fungi and plants with negative influence of the soil carbon accumulation and phosphorous cycling as well as a reduction in the amount of photosynthetic carbon left for fungi. These factors go against crop yield and soil fertility and hence a deeper evaluation is needed in terms of agriculture yields and maintain ecological balance [144]. As discussed earlier regarding the coating of MNPs to make them more stable and compatible, a study used identical size (5–6 nm) IONPs with four different coatings of ascorbate (ASC-IONP), citrate (CIT-IONP), dextran (DEX-IONP) and polyvinyl-pyrrolidone (PVP-IONP) on water flea (*Daphnia magna*). The results stated that each IONP had individual effects but PVP-IONP demonstrated the lowest acute toxicity in comparison to other IONPs with the highest colloidal stability and low rate of accumulation and adsorption. The results from the paper also suggested that toxicity can occur due to a decrease in colloidal stability, release of ions from the core material and formation of ROS, but more importantly they discussed how the negative charge carrier coatings of ASC and CIT-IONP seems to be of lesser importance of being tested in vivo in comparison to in vitro [145]. Along similar lines, when the toxicity of DMSA-coated and unfunctionalized MNPs (fresh and aged uncoated IONP) was tested on three aquatic organisms, namely green algae (*Raphidocelis subcapitata*), duckweed (*Lemna minor*) and water fleas (*Daphnia magna*), the results validated the green algae to be the most sensitive with an EC_50_ of 0.86–2.27 μM at 72 h in comparison to daphnia which displayed minor effects iwith uncoated IONPs over a wide concentration range of 1.0 × 10^−6^ to 100 mg/L [146]. Later in another study, the mechanistic role of these NPs was considered after they were provided orally to *Drosophila* (fruit fly) as a model organism. After entering the gut, these NPs crossed the peritrophic membrane and were able to induce apoptosis. This toxicity within the gut resulted in delays in development, decreased pupa counts and fly hatching with weight loss. After the exposure to nanoparticles when adult flies hatched they showed phenotypic as well as genotypic defects such as sensory organs, other body parts and symptoms of impaired larva crawling or adult climbing, respectively. The alteration in the phenotypic as well as behavioral assay after exposure to NPs was attributed to problems in signaling pathways like Notch, Wnt, and estimated glomerular filtration rate (EGFR) [147]. All these study results demand more data on the basis of cell signaling, proteomics and genomics to observe the underlying mechanisms behind the toxicity.

### 4.4. MNP Toxicity in Vertebrates 

To detect the prenatal exposure of iron oxide MNPs on the basis of dose dependency and surface charge on male and female Crl:CD1(ICR) (CD-1) mice, by exposing them to positively-charged polyethyleneimine-Fe_2_O_3_-NPs (PEI-NPs) and negatively-charged poly (acrylic acid)-Fe_2_O_3_-NPs (PAA-NPs) through intraperitoneal injections at a low and high dose of 10 and 100 mg/kg respectively, observations were performed on gestation days 8, 9 and 10. Consequently, a low dose of MNPs, regardless of charge, did not induce toxicity and high dose exposure led to positive charge-dependent fetal loss as well as morphological alterations of the uteri (both charges) and testes (positive only) of surviving offspring [85]. In another interesting in vivo model studying a chick embryo chorioallantoic membrane model (CAM), Fe_3_O_4_/salicylic acid nanoparticles with different sizes ranging from 60.3 nm and 79.9 nm aqueous dispersion were injected into embryos and after 24 h, the heart, liver, kidney and lungs from embryos were autopsied and prepared for histological analysis. Tissue MNPs deposits were biocompatible with embryos and chickens showing MNPs with 50–100 nm diameter range had no embolic risk after a safe intravenous administration. Also long intravascular persistency showed Fe_3_O_4_/salicylic acid NPs as magnetically good targeting agents [91].

Compared to rodents and chickens, aquatic animals like fish provide a good in vivo model to study the potential toxicity resulting from MNP exposure due to the advantages of easy MNP delivery by waterborne exposure, high biological sample size produced from a single parent to reduce inter-species variation and it can greatly reduce the sacrifice of higher vertebrates to fit the 3R principle. In a study the toxicity of iron oxide nanoparticles was compared with iron salts in blackfish (*Capoeta fusca*). After a series of toxicity studies and chronic exposure to a sub-lethal concentration of Fe_3_O_4_ NPs, and iron salts (ferric nitrate, Fe(NO_3_)_3_; ferric chloride (FeCl_3_) and ferrous sulfate, (FeSO_4_) the authors measured iron uptake over a period of 28 days. The study demonstrated that Fe(NO_3_)_3_ was the most acutely toxic compound, followed by FeCl_3_, FeSO_4_, and Fe_3_O_4_ NPs. Exposure to Fe_3_O_4_ NPs and iron salts caused histopathologic abnormalities in both gills and intestine that included aneurism, hyperplasia, edema, fusion of lamellae, lamellar synechiae, and clear signs of necrosis (in the gills) and increases in the number of goblet cells, blood cell counts, and number of lymphocytes in the intestine cells. Fe_3_O_4_ NPs showed a higher level of uptake in the body tissues compared with iron salts (*p* < 0.05) with levels of Fe in the gill > intestine > liver > kidney. Fe was shown to be eliminated most efficiently from the gills, followed by the kidney, then liver and finally the intestine. The highest tissue bioconcentration factors (BCF) occurred in the liver for FeCl_3_, Fe_3_O_4_ NPs, and FeSO_4_ and in the gills for Fe(NO_3_)_3_ [148].

In a recent study, Malhotra and colleagues discovered that bare Fe_3_O_4_ MNPs (15 nm) exposure can trigger behavioral and biochemical alterations in zebrafish. They incubated adult zebrafish with either low dose (1 ppm) or high dose (10 ppm) bare Fe_3_O_4_ MNPs for 14 days and discovered no significant abnormalities at behavioral and biochemical levels in the low concentration group, whereas, significant changes in aggressiveness, speed, and locomotion behavior coupled with substantial changes in neurotransmitters and stress hormones were induced in the brains when zebrafish were challenged with high dose of Fe_3_O_4_ MNPs [80]. Next, Malhotra and colleagues further tested the idea whether the toxicity of bare Fe_3_O_4_ MNPs can be reduced if their surface was coated with inert substances like carbon. By comprehensive behavioral, biochemical and dimensional reduction assessments, they provided solid evidence to support the notion that surface modification of Fe_3_O_4_ MNPs by carbon coating indeed can increase their biosafety in vivo [103].

In a study MNPs with two distinctive cores of ferrite and manganese ferrite were used to study the toxicity effects in vitro as well as in vivo. Both these MNPs cores were pegylated and two size ranges of 3 nm and between 14–20 nm were generated. 90% cell viability was maintained at a concentration of 50 µg/mL in an in vitro assay in mouse microglia cell line N13., whereas on exposure to zebrafish embryos manganese-based MNPs showed a low survival rate of <50% at a concentration of 100 µg/mL indicating toxicity in comparison to no mortality and normal hatching observed in the case of iron oxide MNPs. Based on these similar physiochemical and magnetic properties, PEGylated iron oxide MNP (20 nm) in cubic shape was selected to be further studied in male Balb/c mice, via tail vein injection at a concentration range of 5 mg/kg body weight. This experiment resulted in no toxicity and good MRI contrast [101]. The screening of MNPs toxicity on inexpensive and pertinent zebrafish embryos model in this study emphasized zebrafish suitability to establish parameters to study on expensive mice model. Along similar lines, green synthesis of MNPs (Fe_2_O_3_) was attained using spinach (*Spinacia oleracea)* leaves of 100–250 nm in size and then zebrafish embryos were exposed to the MNPs 8 hpf–7 dpf at a concentration range of 1, 5, 10, 50 and 100 mg/L. This Fe_2_O_3_ caused a decrease in embryo motility, delays in hatching rate and mortality in the early life stages in zebrafish embryos. The estimated LC_50_ value was 10 mg/L, while most developmental toxicity happened between the 50–100 mg/L concentration range. The authors of the paper suggested NP aggregation as the major cause of toxicity as particles remained undissolved in water, saline and PBS where they can aggregate to obstruct the embryonic chorion pores and stop the exchange of dissolved oxygen and other nutrients [149]. A recent study of MNP toxicity in chicken embryos at a dose range of 10, 25, 50, 100 and 200 µg/mL indicated 100% mortality at 200 µg/mL due to the overloading of Fe^2+^ ions. Decrement in whole weight and crown rump length of chicken embryo at a concentration of 50–100 µg/mL due to NPs-albumin interactions was suggested. Based on the fact that these NPs have the ability to cross the blood brain barrier the histology of brain tissue samples was examined where at 10–100 µg/mL, 50–60% degeneration of neurons was observed. This paper suggested the testing of these NPs into various animal models before coming up with point of reference for use in biomedical applications [150].

In the next study, the toxicity and metabolization of DMSA-, citric acid- and PEG-coated single core and multicore MNPs were analyzed for their toxicological properties. For in vitro analysis, human hepatocellular carcinoma (Hep G2) and human colorectal adenocarcinoma (Caco-2) cells and a amphibian (*Xenopus laevis*) model during its embryo development were used. Up to a concentration of 160 μg/mL, the cell lines remained intact. Neither single core nor multicore MNPs were found to be lethal in vivo, but size-dependent differences in uptake and deposition of the nanoparticles was seen, where single core NPs were absorbed 3–5 fold more for doses of 0.5 and 1 mg/mL in comparison to multicore NPs. The biodegradation of these NPs and metabolization in the early developmental stage is important to understand the toxicity parameters, hence this study suggested vertebrate models like *Xenopus laevis*, for consideration as they may offer quick, inexpensive and high-quantity substitutes prior to toxicity evaluation of nanotherapeutics in rodent models [151]. In another study, the toxicity and biodegradation of ZnxFe_3_O_4_ was studied in vitro on mammalian cell lines (Calu-3, Caco2, Raw 264.7 and MDCK and Hep G2 cell lines) as well as in vivo on a *Xenopus laevis* embryo model. Zn-Fe_3_O_4_ revealed non-toxic results in mammalian cell lines, maintaining cell viability and moderate toxicity in Raw 264.7 and MDCK cell lines at a NP concentration of 1 mg/mL. In the in vivo study, abnormal phenotypes were identified, with swollen and deformed gastrointestinal tracts. At short term exposure of 72 h predominant absorption of NPs was observed, with overexpression of metal response proteins and metal transporters, whereas after long term exposure of 120 h the upregulated genes involved in metal accumulation returned to basal levels for both iron and zinc in the body, which primarily suggests that at the long term exposure stage, the nanoparticle absorption process is much less because of underlying processes of metabolism, distribution and excretion [152].

In another examination, the toxicity of magnetic iron oxide (MION) nanoparticles made along with the carob leaf extract was evaluated, where they were incorporated in certain brain areas of Wistar rats together with carob leaf extracts. Thirty rats were randomly divided into two main groups: control group and MIONs-treated group (15 rats in each group, six rats of each group were used for iron content measurements by inductively coupled plasma-optical emission spectrometry (ICP-OES), while nine rats of each group were used for biochemical analysis and histopathology examination; the right half of each brain sample was used for biochemical analysis while the left half of each brain sample was used for histopathology examination. The rats of the MIONs-treated group received a single i.v. injection with MIONs with a dose of 10 mg/kg. Animals of the control group received an i.v. injection with saline of the same volume as the treated group. Although no remarkable body weight changes were observed, the hippocampus and striatum area of the brain demonstrated neuronal degeneration caused by the magnetic iron oxide nanoparticles. In addition, the magnetic iron oxide nanoparticles also caused disruption of iron heomeostasis in striatum and midbrain by decreasing iron content [153].

In an interesting study to test the biocompatibility of MNPs prostate specific membrane antigen (PSMA)-coated MNPs were conjugated with J591 antibody to the extracellular epitope of PSMA were done to enhance MRI of prostate cancer. In vitro enhanced cellular iron uptake was observed with no effect on prostate cancer cell viability, whereas, specific binding to PSMA-J591 MNP was found when studied in vivo in orthotopic tumor-bearing NOD/SCID mice for 2 h and 24 h via an intravenous injection. An improved MRI contrast for tumors was observed, in turn enhancing the localization/detection of prostate cancer [89]. In another study, intravenous administration of sub-lethal doses (equivalent to 0.1, 1 and 10% of LD_50_) Na-oleate-coated Fe_3_O_4_ (OC-Fe_3_O_4_) NPs on liver structure was detected on female Wistar rats (8 weeks), at a concentration range of 0.0364, 0.364 and 3.64 mg Fe_3_O_4_/kg body weight. The samples were obtained at 24 h, 1, 2 and 4 weeks-post injection for significant effects. In the results some disturbances of liver antioxidant enzymes were observed after 1-week post exposure with glutathione peroxidase (GPx) and glutathione *S*-transferase (GST) being most sensitive, whereas, mitochondrial respiration increased after 2 weeks after injection with 10% OC-Fe_3_O_4_. In the histological sections of liver later, mild necrosis and lipidosis changes in sinusoid space were observed. Overall, the observation in the data suggested that the functional integrity of liver was maintained under sub-lethal doses of OC-Fe_3_O_4_ NPs with mild tissue injury [154]. In another study 10 weeks old pregnant female Wistar mice were used to study the toxicity of MNPs towards neonatal liver at three different embryonic ages using immunohistochemical and histochemical techniques. As drug delivery through vagina for treating neonatal disease is a new technique here MNPs were introduced through the vagina of pregnant mice at days 12, 15 and 17 after fertilization. MNPs in the neonatal liver parenchyma were observed at day 20. MNPs also caused lymphatic infiltration, cytoplasmic vacoulation and mild apoptosis of hepatocytes in the neonatal liver at day 15. Also, increases in production of caspase 3 proteins and tumor necrosis factor receptor-2 (TNFR-2) proteins (indicator of apoptosis), collagen fibers and connective tissue growth factor (CTGF) protein at day 15 were observed, depicting abnormalities caused by MNPs. Overall, the paper concluded mild toxicity of MNPs to the neonatal liver, hypothezing that MNPs can pass in embryo blood or lymphatic circulation and accumulate in embryonic liver [155]. Later on an in vivo analysis of acute toxicity of CoFe_2_O_4_ NPs (40 nm) in zebrafish larvae at a concentration range of 10-500 µM for 96 h, yielded results in a time and dose dependent manner with hatching delay, mechanical damage to membranes, and severe apoptosis of cells in the head, heart and tail region. The biochemical markers studied confirmed ROS-induced acute toxicity with increase in concentration, and inhibition of catalase (CAT) activity. Increase in glutathione-s-transferase (GST) and acid phosphatase (AP) at low concentration of CoFe_2_O_4_ NPs also resulted in oxidative stress [86]. A summary of bare and surface-modified iron oxide MNPs’ in vitro and in vivo toxicity has been compiled in Table 1.

## 5. Conclusions and Future Directions

MNPs have been established for multifunctional applications. They offer an immense possibility of surface modification using various biocompatible, bioactive materials, ligands, and antibodies to name but a few. Following these desirable features MNPs can be targeted, controlled and monitored to optimize the required therapeutically measures. However, toxicity concerns prevent the adoption of MNPs in clinical treatments till now. This gap in the toxicity evaluation is the major concern to collect data and set the safety limits for practical usage of these MNPs in health care industries. Therefore, significant amount of works needs to be analyzed for efficient outcomes both in vitro and in vivo.

An in vitro model for toxicity study is essential for estimation of the amount of dosage and concentration required to be tested further in an in vivo platform to allow correlations between findings. Fe_3_O_4_ MNPs at 25 µg/mL concentration caused damage in vitro THP-1 cells, and in accordance to apoptosis in vivo rat model, at a concentration of 12 mg/kg [79]. Similarly, for in vitro cytotoxicity analysis when a lactate dehydrogenase (LDH) assay was performed in a BeWo b30 placental barrier model, all concentrations of uncoated Fe_3_O_4_ released LDH except 3 µg/cm^2^ after 24 h exposure and LDH levels were elevated from cells treated with uncoated Fe_3_O_4_ at 75 and 15 ug/cm^2^ representing a significant time-dependent increase of toxicity at high concentrations of 15 and 75 µg/cm^2^ for uncoated Fe_3_O_4_, but not for OC-Fe_3_O_4_ MNPs [82]. In a zebrafish system, Malhotra and colleagues also found that carbon-coated Fe_3_O_4_ MNPs displayed superior biosafety compared to uncoated ones based on neurobehavioral and biochemical assays [80]. All these phenomena indicate that a lot of factors are involved in understanding the toxicity on a cellular and organism basis depending on dosage, concentration, time, surface chemistry, cell type, interaction medium, internalization mode, which need very elaborate studies to understand the fundamental mechanism properly in accordance to be able to use the MNPs in healthcare. The contradictions amongst in vitro and in vivo findings might be attributed to the issue of MNP surface modification. Different studies have suggested the use of intended biomaterials declaring safe them for an in vivo usage. An in vitro study can clarify the uptake, toxicity, cell medium interaction and cell-moment analysis of MNP up to a level of single cell track precision, and therefore the relevant parameters can be mimicked in in vivo studies to a closer extent for successful results and conclusions.

Toxicity can arise due to different means, through toxicity of the precursors used for MNP preparation, through disturbances of the ongoing cellular mechanisms and through the normal functioning of the body cycle inside an organism. In previous studies it has been observed that similar MNPs can pose different toxicities in different study models. To better understand the underlying mechanism, multi-omics studies using proteomics, genomics and metabolomics are required which can help assess the toxicity of these MNPs at precise levels. The better usage and safety limits of MNPs are very important to understand their fate, performance and toxicity. Therefore, in this review we have collected the research from the latest development in the field of evaluation of MNPs toxicity and conclude that a lot more studies are still required to be done, to bring light to this topic with reliable data for usage in practical medical applications. Also, approaches like metabolomics, proteomics and genetics studies should be performed simultaneously to understand the basis of differences in cytotoxicity in different cell lines and organisms. It is also worth noting that this review article is largely focused on an eco-nanotoxicological point of view. Further reviews to distinguish between the toxicity of nanoparticles administered in a precise manner to exert a therapeutic or diagnostic effect, from the toxicity derived from accidental exposition of the animal should be addressed.

## Figures and Tables

**Table 1:** Summary of iron oxide magnetic nanoparticle (MNPS) toxicity. Extensive amount of work is going on in area of toxicity studies of MNPs in various different combinations with appropriate enhancement with ligands, antibodies, polymeric coating, green synthesis, infusion of drug, hyperthermia application and external magnetic control with retention in superparamagnetism to be able to detect and direct MNPs at desired location. With many papers coming up each year in the field we have compiled a list of papers from year 2015–2019 to indicated the different types of (iron oxide) MNPs used in different kind of models recently to study the toxicological response of these MNPs.

Type of MNPs	Size and Shape of Tested MNPs	Model Organism (In Vitro or in Vivo Test)	Method of Toxicity Analysis	Treatment Condition (Time and Dose)	Results	Ref.
**Uncoated Magnetic Nanoparticles (MNPs)**						
**Bare Fe_3_O_4_-MNPs**	72.6 ± 0.6 nm spheroid	THP-1 cells and female CD(R) IGS rats	Biochemical marker in rat blood after treatment	In vitro: 100, 800 and 1600 μg/mL 24 h In vivo: 12 mg/kg/intravenous injection 6 days	Fe_3_O_4_-MNPs cytotoxicity in erythrocytes in vitro and in vivo	[79]
	15 nm	Adult zebrafish	Behavioral and biochemical assessment in adult zebrafish	14 days waterborne incubation at 1 and 10 ppm	Uncoated MNPs exhibited behavior and biochemical safety at 1ppm but display neurobehavioral toxicity at 10 ppm	[80]
	15 nm and 225 nm spherical	A549 cells and Male Balb/c mice	Cell viability assay	In vitro:10–80 μg/mL In vivo: Subcutaneous injection of 2 × 10^6^ cells suspended in 100 µL PBS	Magnetic nanomaterials did not indicate inherent toxicity	[81]
**Surface coated/modified MNPs**						
**(OC-Fe_3_O_4_) NPs** **(Fl-SiO_2_)**	8 nm, 25 nm and 50 nm	BeWo b30 placental barrier model	Lactase dehydrogenase (LDH) in cell culture	4, 24 or 48 h 75, 15, 3, 0.6 and 0.12 µg/cm^2^	Iron oxide MNPS triggers cytotoxicity at lower doses and shorter exposure compared with silica NPs	[82]
**CSO-INPs**	6 ± 1.2 nm 8 ± 2.7 nm	HeLa, A549 and HeK293 cells	MTT assay	24, 48 and 72 h 0.5, 2, 4 μg/µL	INPs triggers toxic effects in Hek293, A549 and Hela cells in comparison to CSO-INPs	[83]
**L14@Fe_3_O_4_** **L4@Fe_3_O_4_** **Gly@Fe_3_O_4_**	11 ± 3 nm 7 ± 2 nm 9 ± 2 nm spherical	HeP G2 cells	MTT assay	24 and 48 h 1–500 μg/mL	Cytotoxicity of naked SPION increased in relation to increasing concentration	[84]
**Fe_2_O_3_-NPs** **PEI-NPs** **PAA-NPs**	28–30 nm	Male and female Crl:CD1(ICR) (CD-1) mice	Dams: gestation period of toxicity Cesarean: Histopathology analysis	Gestation day 8, 9, or 10 low dose:10 mg/kg high dose:100 mg/kg	A low dose of NPs, regardless of charge, did not induce toxicity; high exposure led to charge-dependent fetal loss	[85]
**(HLC) Fe_3_O_4_ NPs**	8.4 nm spherical	NIH3T3 cells	FluoStar Optima microplate reader	24 h 25 to 250 μg/mL	Reduced toxicity towards normal cells, enhancing the potential of magnetic hyperthermia in cancer treatment	[86]
**DMSA-SPION**	15 nm	MCF-7 cells	MTT assay Trypan blue exclusion test	1 h–72 h 0.4 mg/mL	MCF-7 accumulated NPs without effect on cell morphology, ROS generation and cell viability	[87]
**Dox-gold coated MNPs** **MGNPs-DOX-M-group**	MNP: 10 nm MGNPs: 22 nm spherical	Ehrlich ascites carcinoma cells injected intraperitoneally into female Balb/c mice	Histological examination Tumor size (AST, ALT, CK-MB, LDH)	20 mice group 10 mg/kg/group external application of neodymium–iron–boron magnetic disc (1.14 T) at tumor site for 3 h	Best therapeutic anti-cancer activity and lowest systemic toxicity compared to free DOX	[88]
**PLGA NPs sorafenib SPION** **SRF/FA-PEG-PLGA NP**	205 ± 3 nm spherical	BEL7402 cancer cells	MTT assay Apoptosis assay Anticancer efficacy	72 h 10 and 40 mg iron/mL	Concentration dependent cytotoxicity in BEL7402 cancer cells	[89]
**Starch- Fe_3_O_4_ MNPs** **Dextran-Fe_3_O_4_ MNPs**	100 nm	Rat PC 12 cells (ATCC)	Cell-viability assay	1 h–72 h 0.01–0.5 mg/mL	Uncoated- Fe_3_O_4_ MNPs maximum interaction and entered inside cell with no cytotoxic effect	[90]
**Fe_3_O_4_/salicylic acid NPs**	MNPs 33–277.9 nm Embryos injected: 60.3 nm and 79.9 nm MNPs	chick embryo chorioallantoic membrane model (CAM)	Morphological analysis	24 h Autopsied to harvest embryo viscera (heart, kidney, liver, and lung). 0.15 mL MNPs	50–100 nm diameter range MNPs had no embolic risk, on a safety intravenous administration. Tissue MNPs deposits were biocompatible with embryos and chicken	[91]
**PEI-MNP**	Not available	Human neuroblastoma SH-SY5Y cells (ATCC CRL-2266)	Quantitative/qualitative flow cytometry of apoptosis and necrosis	External hyperthermia (EHT), Magnetic hyperthermia (MHT)	A maximum difference in cytotoxicity approximately 45% was observed at T_0_ = 46 °C.	[92]
**AA coated IONPs**	3.98, 4.09, 3.41, 4.32, 2.35 nm globular	HFF2 cell lines	MTT assay	72 h 0.049, 0.073, 0.110, 0.165, 0.248 and 0.373 mg/mL	IONPs were biocompatible and nontoxic with the cell line HFF2	[93]
**Multifunctional MNPs** **Anti-CD47 antibody** **Gemcitabine**	109 ± 1 nm	CD47-positive pancreatic cancer cells	Resazurin dye	24 h Free Gem (0.1, 0.4 and 1 µM) MNP-Gem and MNP Gem-anti-CD47 (0.2 mg Fe/mL, 4.8 µM Gem, Ab 20 μg/mg Fe)	Cytotoxic activity of the multifunctional Nano formulation is not increased in the in vitro studies	[94]
**Rosi-MNPs** **Al-MNPs** **Un-MNPs**	21 ± 4 nm	Magnet and Sham mice	MTT assay	24 h 48 h 0.5, 5, 50, and 500 μg/mL	Al-MNPs only caused a significant reduction in cell viability at 500 μg/mL	[95]
**MTX** **F-Lys-MTX NPs**	43.72 ± 4.73 nm	MCF-7 cell lines	MTT assay	48 and 72 h 100 mL	MTX-conjugated NPs: reduction in cellular viability in human breast cancer (MCF-7) cells compared to free MTX over time	[96]
**F@Tyr NPs** **F@Tyr@TMX NPs**	22.19 ± 3.58 nm	HEK-293 MCF-7 cells	Hemolysis test and MTT assays	72 h F@Tyr NPs, Bare Fe_3_O_4_ 0.025, 0.05, 0.1, 0.2, 0.4 and 0.8 mg/mL	Cytotoxicity study, F@Tyr@TMX NPs exhibited more cytotoxic effects than free TMX	[96]
**IONPs-PEG** **IONPs-PEI** **SEI-10** **SMG-10** **SMG-30**	10–30 nm	SKOV-3 RAW 264.7 Nude mice BALB/c mice	LDH assay, Hemolysis, ROS, MMP Cell cycle analysis, in vivo bio-distribution, toxicity	Hemolysis: 200 µL, 4 h. In vivo biodistribution: dose of 1.5 mg Fe/kg. In vivo toxicity: 1.5, 2.5, or 5 mg/kg	No obvious toxicity was found for PEGylated IONPs in BALB/c mice, whereas PEI-coated IONPs exhibited dose-dependent lethal toxicity	[97]
**F@BSA@CURNPs**	56 ± 11.43 nm, spherical	HFF2 MCF-7 cells	Cell viability by MTT assay	72 and 96 h Serial dilution 15–950 µM	F@BSA@CUR NPs had much higher cytotoxicity against MCF7 cells	[98]
**CS-DX-SPIONs**	55 nm round shape	In vitro: Rat C6 glioma, human U87 glioma, and human cervix carcinoma HeLa cells and Male Wistar rats	Histology analysis	24 h In vitro: 1, 10, 50, and 150 μg/mL 1, 3, 6, 12 intravenous injections of PBS via tail vein; DX-SPIONs (Fe concentration of 2.5 mg/kg); CS-DX-SPIONs (Fe at 2.5 mg/kg).	Increase in surface charge of the NPs due to the chitosan coating enhanced the intracellular uptake of particles and thus increased their cytotoxic activity.	[99]
**Asparaginase enzyme-immobilized on APTES modified MNPs**	50–100 nm	In vitro: Reduction of acrylamide in food model system	Deactivation rate constant (K_d_) of free and immobilized enzyme	Five cycles of pretreatment	It was found to be more than three-fold increase their thermal stability from free enzyme and retained 90% activity after fifth cycle	[100]
**MnFe_2_O_4_** **MnFe1** **MnFe2**	3–20 nm	Mouse microglial cell line N13 and Zebrafish embryos Male Balb/c mice	Teratogenicity assay	In vitro: 0.1 to 100 μg/mL In vivo: 0.01, 0.1, 1, 10, 100 μg/mL In vivo: Fe 1, Fe 2. PEGylated Cubic (20 nm)	No significant cytotoxicity, till 24 h; No mortality or malformations were observed in the embryos exposed to different doses of particles at 48 hpf. At 100 μg/mL high percentage of mortality 6 dpf	[101]
***n*-octyltriethoxysilane coated-MNPs**	17.9 ± 3.9 nm 18.7 ± 4.4 nm	PC12 and ReN cell VM	Cell Viability LIVE/DEAD Staining Prussian Blue and Nuclear Fast Red Staining	24 h 4, 8, 16, and 32 µg	Coated MNPs decreased cytotoxic effects; Significant differences in toxicological profiles in two mammalian cell lines	[102]
**Carbon-coated MNPs**	24 nm	Adult zebrafish	Multiple behavioral and biochemical tests	1 and 10 ppm exposure for 14 days	Carbon-coated MNPs can significantly enhance its biosafety by reducing neurobehavioral toxicities compared to the bare MNPs	[103]

## References

[1] Feynman R.P. (1960). There’s plenty of room at the bottom. Calif. Inst. Technol. Eng. Sci. Mag..

[2] Park S., Lim J., Kim J., Yun H., Kim C. (2006). Toxicity estimation of magnetic fluids in a biological test. J. Magn. Magn. Mater..

[3] Anselmo A.C., Mitragotri S. (2019). Nanoparticles in the clinic: An update. Bioeng. Transl. Med..

[4] Harisinghani M.G., Barentsz J., Hahn P.F., Deserno W.M., Tabatabaei S., van de Kaa C.H., de la Rosette J., Weissleder R. (2003). Noninvasive detection of clinically occult lymph-node metastases in prostate cancer. N. Engl. J. Med..

[5] Agency E.M. (2008). Withdrawal Assessment Report for Sinerem.

[6] Wang Y.-X.J. (2015). Current status of superparamagnetic iron oxide contrast agents for liver magnetic resonance imaging. World J. Gastroenterol..

[7] Varallyay C.G., Toth G.B., Fu R., Netto J.P., Firkins J., Ambady P., Neuwelt E.A. (2017). What does the boxed warning tell us? Safe practice of using ferumoxytol as an mri contrast agent. Am. J. Neuroradiol..

[8] Auerbach M., Chertow G.M., Rosner M. (2018). Ferumoxytol for the treatment of iron deficiency anemia. Expert Rev. Hematol..

[9] Coricovac D.-E., Moacă E.-A., Pinzaru I., Cîtu C., Soica C., Mihali C.-V., Păcurariu C., Tutelyan V.A., Tsatsakis A., Dehelean C.-A. (2017). Biocompatible colloidal suspensions based on magnetic iron oxide nanoparticles: Synthesis, characterization and toxicological profile. Front. Pharmacol..

[10] Conde J., Dias J.T., Grazú V., Moros M., Baptista P.V., de la Fuente J.M. (2014). Revisiting 30 years of biofunctionalization and surface chemistry of inorganic nanoparticles for nanomedicine. Front. Chem..

[11] Soares P.I., Laia C.A., Carvalho A., Pereira L.C., Coutinho J.T., Ferreira I.M., Novo C.M., Borges J.P. (2016). Iron oxide nanoparticles stabilized with a bilayer of oleic acid for magnetic hyperthermia and mri applications. Appl. Surf. Sci..

[12] Medeiros S.F., Filizzola J.O., Fonseca V.F., Oliveira P.F., Silva T.M., Elaissari A., Santos A.M. (2015). Synthesis and characterization of stable aqueous dispersion of functionalized double-coated iron oxide nanoparticles. Mater. Lett..

[13] Valdiglesias V., Fernandez-Bertolez N., Kiliç G., Costa C., Costa S., Fraga S., Bessa M.J., Pasaro E., Teixeira J.P., Laffon B. (2016). Are iron oxide nanoparticles safe? Current knowledge and future perspectives. J. Trace Elem. Med. Biol..

[14] Tran N., Webster T.J. (2010). Magnetic nanoparticles: Biomedical applications and challenges. J. Mater. Chem..

[15] Shete P., Patil R., Tiwale B., Pawar S. (2015). Water dispersible oleic acid-coated fe3o4 nanoparticles for biomedical applications. J. Magn. Magn. Mater..

[16] Tran T.T.-D., Van Vo T., Tran P.H.-L. (2015). Design of iron oxide nanoparticles decorated oleic acid and bovine serum albumin for drug delivery. Chem. Eng. Res. Des..

[17] Gupta A.K., Gupta M. (2005). Synthesis and surface engineering of iron oxide nanoparticles for biomedical applications. Biomaterials.

[18] Sun J., Zhou S., Hou P., Yang Y., Weng J., Li X., Li M. (2007). Synthesis and characterization of biocompatible fe3o4 nanoparticles. J. Biomed. Mater. Res. Part A.

[19] Sun C., Sze R., Zhang M. (2006). Folic acid-peg conjugated superparamagnetic nanoparticles for targeted cellular uptake and detection by mri. J. Biomed. Mater. Res. Part A Off. J. Soc. Biomater. Jpn. Soc. Biomater Aust. Soc. Biomater. Korean Soc. Biomater..

[20] Hirsch L.R., Stafford R.J., Bankson J.A., Sershen S.R., Rivera B., Price R., Hazle J.D., Halas N.J., West J.L. (2003). Nanoshell-mediated near-infrared thermal therapy of tumors under magnetic resonance guidance. Proc. Natl. Acad. Sci. USA.

[21] Arbab A.S., Bashaw L.A., Miller B.R., Jordan E.K., Lewis B.K., Kalish H., Frank J.A. (2003). Characterization of biophysical and metabolic properties of cells labeled with superparamagnetic iron oxide nanoparticles and transfection agent for cellular mr imaging. Radiology.

[22] Bulte J.W., Kraitchman D.L. (2004). Iron oxide mr contrast agents for molecular and cellular imaging. NMR Biomed. Internatl. J. Devot. Dev. Appl. Magn. Res. Vivo.

[23] Pardoe H., Chua-Anusorn W., Pierre T.G.S., Dobson J. (2001). Structural and magnetic properties of nanoscale iron oxide particles synthesized in the presence of dextran or polyvinyl alcohol. J. Magn. Magn. Mater..

[24] Petri-Fink A., Steitz B., Finka A., Salaklang J., Hofmann H. (2008). Effect of cell media on polymer coated superparamagnetic iron oxide nanoparticles (spions): Colloidal stability, cytotoxicity, and cellular uptake studies. Eur. J. Pharm. Biopharm..

[25] Józefczak A., Hornowski T., Skumiel A., Závišová V., Koneracká M., Tomašovičová N., Timko M., Kopčanský P., Kelani H. (2012). Effect of the molecular weight of poly (ethylene glycol) on the properties of biocompatible magnetic fluids. Internatl. J. Thermophys..

[26] Soleymani M., Edrissi M. (2016). Synthesis of bilayer surfactant-coated magnetic nanoparticles for application in magnetic fluid hyperthermia. J. Dispers. Sci. Technol..

[27] Mamalis A. (2007). Recent advances in nanotechnology. J. Mater. Process. Technol..

[28] Oberdörster G., Oberdörster E., Oberdörster J. (2005). Nanotoxicology: An emerging discipline evolving from studies of ultrafine particles. Environ. Health Perspect..

[29] Srivastava V., Gusain D., Sharma Y.C. (2015). Critical review on the toxicity of some widely used engineered nanoparticles. Ind. Eng. Chem. Res..

[30] Sharma Y.C., Srivastava V., Singh V., Kaul S., Weng C. (2009). Nano-adsorbents for the removal of metallic pollutants from water and wastewater. Environ. Technol..

[31] Zeng W., Gao L., Guo J. (1998). A new sol-gel route using inorganic salt for synthesizing al2o3 nanopowders. Nanostruct. Mater..

[32] Arruebo M., Fernández-Pacheco R., Ibarra M.R., Santamaría J. (2007). Magnetic nanoparticles for drug delivery. Nano Today.

[33] Vallabani N.S., Singh S. (2018). Recent advances and future prospects of iron oxide nanoparticles in biomedicine and diagnostics. 3 Biotech.

[34] Mohammed L., Gomaa H.G., Ragab D., Zhu J. (2017). Magnetic nanoparticles for environmental and biomedical applications: A review. Particuology.

[35] McBain S.C., Yiu H.H., Dobson J. (2008). Magnetic nanoparticles for gene and drug delivery. Int. J. Nanomed..

[36] Pankhurst Q.A., Connolly J., Jones S., Dobson J. (2003). Applications of magnetic nanoparticles in biomedicine. J. Phys. D Appl. Phys..

[37] Carpenter E.E. (2001). Iron nanoparticles as potential magnetic carriers. J. Magn. Magn. Mater..

[38] Koneracka M., Kopčanský P., Timko M., Ramchand C., De Sequeira A., Trevan M. (2002). Direct binding procedure of proteins and enzymes to fine magnetic particles. J. Mol. Catal. B Enzym..

[39] Wu W., He Q., Jiang C. (2008). Magnetic iron oxide nanoparticles: Synthesis and surface functionalization strategies. Nanoscale Res. Lett..

[40] Sun S., Zeng H. (2002). Size-controlled synthesis of magnetite nanoparticles. J. Am. Chem. Soc..

[41] Fatima H., Lee D.-W., Yun H.J., Kim K.-S. (2018). Shape-controlled synthesis of magnetic fe 3 o 4 nanoparticles with different iron precursors and capping agents. RSC Adv..

[42] Kalantari K., Ahmad M.B., Shameli K., Hussein M.Z.B., Khandanlou R., Khanehzaei H. (2014). Size-controlled synthesis of fe3o4 magnetic nanoparticles in the layers of montmorillonite. J. Nanomater..

[43] Xie W., Guo Z., Gao F., Gao Q., Wang D., Liaw B.-s., Cai Q., Sun X., Wang X., Zhao L. (2018). Shape-, size-and structure-controlled synthesis and biocompatibility of iron oxide nanoparticles for magnetic theranostics. Theranostics.

[44] Li W., Lee S.S., Wu J., Hinton C.H., Fortner J.D. (2016). Shape and size controlled synthesis of uniform iron oxide nanocrystals through new non-hydrolytic routes. Nanotechnology.

[45] Agostini P., Meffre A., Lacroix L.-M., Ugnati D., Ondarçuhu T., Respaud M., Lassagne B. (2016). Electrospray deposition of isolated chemically synthesized magnetic nanoparticles. J. Nanopart. Res..

[46] Akbarzadeh A., Samiei M., Davaran S. (2012). Magnetic nanoparticles: Preparation, physical properties, and applications in biomedicine. Nanoscale Res. Lett..

[47] Bomatí-Miguel O., Mazeina L., Navrotsky A., Veintemillas-Verdaguer S. (2008). Calorimetric study of maghemite nanoparticles synthesized by laser-induced pyrolysis. Chem. Mater..

[48] Majidi S., Zeinali Sehrig F., Farkhani S.M., Soleymani Goloujeh M., Akbarzadeh A. (2016). Current methods for synthesis of magnetic nanoparticles. Artif. Cells Nanomed. Biotechnol..

[49] Roh Y., Vali H., Phelps T., Moon J.-W. (2006). Extracellular synthesis of magnetite and metal-substituted magnetite nanoparticles. J. Nanosci. Nanotechnol..

[50] Bharde A.A., Parikh R.Y., Baidakova M., Jouen S., Hannoyer B., Enoki T., Prasad B., Shouche Y.S., Ogale S., Sastry M. (2008). Bacteria-mediated precursor-dependent biosynthesis of superparamagnetic iron oxide and iron sulfide nanoparticles. Langmuir.

[51] Enriquez-Navas P.M., Garcia-Martin M.L. (2012). Application of inorganic nanoparticles for diagnosis based on mri. Frontiers of Nanoscience.

[52] Hofmann-Amtenbrink M., Hofmann H., Montet X. (2010). Superparamagnetic nanoparticles–a tool for early diagnostics. Swiss Med. Wkly..

[53] Kim J.-E., Shin J.-Y., Cho M.-H. (2012). Magnetic nanoparticles: An update of application for drug delivery and possible toxic effects. Arch. Toxicol..

[54] Lu A.H., Salabas E.E.L., Schüth F. (2007). Magnetic nanoparticles: Synthesis, protection, functionalization, and application. Angew. Chem. Int. Edit..

[55] Wang M., Guo L., Sun H. (2019). Manufacture of biomaterials. Ref. Modul. Biomed. Sci. Encycl. Biomed. Eng..

[56] Liechty W.B., Kryscio D.R., Slaughter B.V., Peppas N.A. (2010). Polymers for drug delivery systems. Ann. Rev. Chem. Biomol. Eng..

[57] Gupta A.K., Naregalkar R.R., Vaidya V.D., Gupta M. (2007). Recent advances on surface engineering of magnetic iron oxide nanoparticles and their biomedical applications. Nanomedicine.

[58] Ma Z., Liu H. (2007). Synthesis and surface modification of magnetic particles for application in biotechnology and biomedicine. China Part..

[59] Shapiro E.M. (2015). Biodegradable, polymer encapsulated, metal oxide particles for mri-based cell tracking. Magn. Res. Med..

[60] Senthilnathan B., Ameerkhan H., Aswini S., Abirami M., Bharath T., Ajithkumar T., Maheswaran A. (2015). Review on various approaches on preparation, characterisation and applications of polymeric nanoparticles. World J. Pharm. Res..

[61] Srivastava A., Yadav T., Sharma S., Nayak A., Kumari A.A., Mishra N. (2015). Polymers in drug delivery. J. Biosci. Med..

[62] Shubayev V.I., Pisanic T.R., Jin S. (2009). Magnetic nanoparticles for theragnostics. Adv. Drug Deliv. Rev..

[63] Kumari A., Yadav S.K., Yadav S.C. (2010). Biodegradable polymeric nanoparticles based drug delivery systems. Coll. Surf. B Biointerfaces.

[64] Wischke C., Schwendeman S.P. (2008). Principles of encapsulating hydrophobic drugs in pla/plga microparticles. Int. J. Pharm..

[65] Martinho N., Damgé C., Reis C.P. (2011). Recent advances in drug delivery systems. J. Biomater. Nanobiotechnol..

[66] Aguilar-Arteaga K., Rodriguez J., Barrado E. (2010). Magnetic solids in analytical chemistry: A review. Anal. Chim. Acta.

[67] Carregal-Romero S., Caballero-Díaz E., Beqa L., Abdelmonem A.M., Ochs M., Hühn D., Suau B.S., Valcarcel M., Parak W.J. (2013). Multiplexed sensing and imaging with colloidal nano-and microparticles. Annu. Rev. Anal. Chem..

[68] Cole A.J., Yang V.C., David A.E. (2011). Cancer theranostics: The rise of targeted magnetic nanoparticles. Trends Biotechnol..

[69] Colombo M., Carregal-Romero S., Casula M.F., Gutiérrez L., Morales M.P., Böhm I.B., Heverhagen J.T., Prosperi D., Parak W.J. (2012). Biological applications of magnetic nanoparticles. Chem. Soc. Rev..

[70] Reddy L.H., Arias J.L., Nicolas J., Couvreur P. (2012). Magnetic nanoparticles: Design and characterization, toxicity and biocompatibility, pharmaceutical and biomedical applications. Chem. Rev..

[71] Zhang Y., Kohler N., Zhang M. (2002). Surface modification of superparamagnetic magnetite nanoparticles and their intracellular uptake. Biomaterials.

[72] Leung K.C.F., Xuan S. (2016). Noble metal-iron oxide hybrid nanomaterials: Emerging applications. Chem. Rec..

[73] Chak C.-P., Xuan S., Mendes P.M., Yu J.C., Cheng C.H., Leung K.C.-F. (2009). Discrete functional gold nanoparticles: Hydrogen bond-assisted synthesis, magnetic purification, supramolecular dimer and trimer formation. ACS Nano.

[74] Chen S.-S., Xu H., Xu H.-J., Yu G.-J., Gong X.-L., Fang Q.-L., Leung K.C.-F., Xuan S.-H., Xiong Q.-R. (2015). A facile ultrasonication assisted method for fe 3 o 4@ sio 2-ag nanospheres with excellent antibacterial activity. Dalton Trans..

[75] Duguet E., Vasseur S., Mornet S., Devoisselle J.-M. (2006). Magnetic nanoparticles and their applications in medicine. Nanomedicine.

[76] Nel A., Xia T., Mädler L., Li N. (2006). Toxic potential of materials at the nanolevel. Science.

[77] Donaldson K., Stone V., Tran C., Kreyling W., Borm P.J. (2004). Nanotoxicology. Occup. Environ. Med..

[78] Unfried K., Albrecht C., Klotz L.-O., Von Mikecz A., Grether-Beck S., Schins R.P. (2007). Cellular responses to nanoparticles: Target structures and mechanisms. Nanotoxicology.

[79] Ran Q., Xiang Y., Liu Y., Xiang L., Li F., Deng X., Xiao Y., Chen L., Chen L., Li Z. (2015). Eryptosis indices as a novel predictive parameter for biocompatibility of fe 3 o 4 magnetic nanoparticles on erythrocytes. Sci. Rep..

[80] Malhotra N., Chen J.-R., Sarasamma S., Audira G., Siregar P., Liang S.-T., Lai Y.-H., Lin G.-M., Ger T.-R., Hsiao C.-D. (2019). Ecotoxicity assessment of fe3o4 magnetic nanoparticle exposure in adult zebrafish at an environmental pertinent concentration by behavioral and biochemical testing. Nanomaterials.

[81] Shen S., Wang S., Zheng R., Zhu X., Jiang X., Fu D., Yang W. (2015). Magnetic nanoparticle clusters for photothermal therapy with near-infrared irradiation. Biomaterials.

[82] Correia Carreira S., Walker L., Paul K., Saunders M. (2015). The toxicity, transport and uptake of nanoparticles in the in vitro bewo b30 placental cell barrier model used within nanotest. Nanotoxicology.

[83] Shukla S., Jadaun A., Arora V., Sinha R.K., Biyani N., Jain V. (2015). In vitro toxicity assessment of chitosan oligosaccharide coated iron oxide nanoparticles. Toxicol. Rep..

[84] Gholami A., Rasoul-amini S., Ebrahiminezhad A., Seradj S.H., Ghasemi Y. (2015). Lipoamino acid coated superparamagnetic iron oxide nanoparticles concentration and time dependently enhanced growth of human hepatocarcinoma cell line (hep-g2). J. Nanomater..

[85] Di Bona K., Xu Y., Gray M., Fair D., Hayles H., Milad L., Montes A., Sherwood J., Bao Y., Rasco J. (2015). Short-and long-term effects of prenatal exposure to iron oxide nanoparticles: Influence of surface charge and dose on developmental and reproductive toxicity. Int. Ernational J. Mol. Sci..

[86] Ahmad F., Liu X., Zhou Y., Yao H. (2015). An in vivo evaluation of acute toxicity of cobalt ferrite (CoFe2O4) nanoparticles in larval-embryo Zebrafish (Danio rerio). Aquat. Toxicol..

[87] Calero M., Chiappi M., Lazaro-Carrillo A., Rodríguez M.J., Chichón F.J., Crosbie-Staunton K., Prina-Mello A., Volkov Y., Villanueva A., Carrascosa J.L. (2015). Characterization of interaction of magnetic nanoparticles with breast cancer cells. J. Nanobiotechnol..

[88] Elbialy N.S., Fathy M.M., Khalil W.M. (2015). Doxorubicin loaded magnetic gold nanoparticles for in vivo targeted drug delivery. Int. J. Pharm..

[89] Tse B.W.-C., Cowin G.J., Soekmadji C., Jovanovic L., Vasireddy R.S., Ling M.-T., Khatri A., Liu T., Thierry B., Russell P.J. (2015). PSMA-targeting iron oxide magnetic nanoparticles enhance MRI of preclinical prostate cancer. Nanomedicine.

[90] Marcus M., Karni M., Baranes K., Levy I., Alon N., Margel S., Shefi O. (2016). Iron oxide nanoparticles for neuronal cell applications: Uptake study and magnetic manipulations. J. Nanobiotechnol..

[91] Buteică S., Mihaiescu D., Rogoveanu I., Mărgăritescu D., Mîndrilă I. (2016). Chick chorioallantoic membrane model as a preclinical tool for nanoparticles biology study. Rom. Biotechnol. Lett..

[92] Sanz B., Calatayud M.P., Torres T.E., Fanarraga M.L., Ibarra M.R., Goya G.F. (2017). Magnetic hyperthermia enhances cell toxicity with respect to exogenous heating. Biomaterials.

[93] Nosrati H., Salehiabar M., Attari E., Davaran S., Danafar H., Manjili H.K. (2018). Green and one-pot surface coating of iron oxide magnetic nanoparticles with natural amino acids and biocompatibility investigation. Appl. Organomet. Chem..

[94] Trabulo S., Aires A., Aicher A., Heeschen C., Cortajarena A.L. (2017). Multifunctionalized iron oxide nanoparticles for selective targeting of pancreatic cancer cells. Biochim. Biophys. Acta (BBA)-Gen. Subj..

[95] Saatchi K., Tod S.E., Leung D., Nicholson K.E., Andreu I., Buchwalder C., Schmitt V., Häfeli U.O., Gray S.L. (2017). Characterization of alendronic-and undecylenic acid coated magnetic nanoparticles for the targeted delivery of rosiglitazone to subcutaneous adipose tissue. Nanomed. Nanotechnol. Biol. Med..

[96] Nosrati H., Rashidi N., Danafar H., Manjili H.K. (2018). Anticancer activity of tamoxifen loaded tyrosine decorated biocompatible fe 3 o 4 magnetic nanoparticles against breast cancer cell lines. J. Inorg. Organomet. Polym. Mater..

[97] Feng Q., Liu Y., Huang J., Chen K., Huang J., Xiao K. (2018). Uptake, distribution, clearance, and toxicity of iron oxide nanoparticles with different sizes and coatings. Sci. Rep..

[98] Nosrati H., Sefidi N., Sharafi A., Danafar H., Manjili H.K. (2018). Bovine serum albumin (bsa) coated iron oxide magnetic nanoparticles as biocompatible carriers for curcumin-anticancer drug. Bioorg. Chem..

[99] Shevtsov M., Nikolaev B., Marchenko Y., Yakovleva L., Skvortsov N., Mazur A., Tolstoy P., Ryzhov V., Multhoff G. (2018). Targeting experimental orthotopic glioblastoma with chitosan-based superparamagnetic iron oxide nanoparticles (cs-dx-spions). Int. J. Nanomed..

[100] Alam S., Ahmad R., Pranaw K., Mishra P., Khare S.K. (2018). Asparaginase conjugated magnetic nanoparticles used for reducing acrylamide formation in food model system. Bioresour. Technol..

[101] Caro C., Egea-Benavente D., Polvillo R., Royo J.L., Leal M.P., García-Martín M.L. (2019). Comprehensive toxicity assessment of pegylated magnetic nanoparticles for in vivo applications. Coll. Surf. B Biointerfaces.

[102] Ma W., Gehret P.M., Hoff R.E., Kelly L.P., Suh W.H. (2019). The investigation into the toxic potential of iron oxide nanoparticles utilizing rat pheochromocytoma and human neural stem cells. Nanomaterials.

[103] Malhotra N., Audira G., Chen J.-R., Siregar P., Hsu H.-S., Lee J.-S., Ger T.-R., Hsiao C.-D. (2020). Surface modification of magnetic nanoparticles by carbon-coating can increase its biosafety: Evidences from biochemical and neurobehavioral tests in zebrafish. Molecules.

[104] Kwon J.-T., Hwang S.-K., Jin H., Kim D.-S., Minai-Tehrani A., Yoon H.-J., Choi M., Yoon T.-J., Han D.-Y., Kang Y.-W. (2008). Body distribution of inhaled fluorescent magnetic nanoparticles in the mice. J. Occup. Health.

[105] Jain T.K., Reddy M.K., Morales M.A., Leslie-Pelecky D.L., Labhasetwar V. (2008). Biodistribution, clearance, and biocompatibility of iron oxide magnetic nanoparticles in rats. Mol. Pharm..

[106] Cole A.J., David A.E., Wang J., Galbán C.J., Yang V.C. (2011). Magnetic brain tumor targeting and biodistribution of long-circulating peg-modified, cross-linked starch-coated iron oxide nanoparticles. Biomaterials.

[107] Wang X., Zhang J., Yang X., Tang Z., Hu Y., Chen B., Tang J. (2014). In vivo assessment of hepatotoxicity, nephrotoxicity and biodistribution using 3-aminopropyltriethoxysilane-coated magnetic nanoparticles (apts-mnps) in icr mice. Chin. Sci. Bull..

[108] Markides H., Rotherham M., El Haj A. (2012). Biocompatibility and toxicity of magnetic nanoparticles in regenerative medicine. J. Nanomater..

[109] Kumamoto Y., Camporez J.P.G., Jurczak M.J., Shanabrough M., Horvath T., Shulman G.I., Iwasaki A. (2016). Cd301b+ mononuclear phagocytes maintain positive energy balance through secretion of resistin-like molecule alpha. Immunity.

[110] O’Keefe J.H., Bhatti S.K., Bajwa A., DiNicolantonio J.J., Lavie C.J. (2014). Alcohol and cardiovascular health: The dose makes the pois or the remedy. Mayo Clin. Proc..

[111] Huang D.-M., Chung T.-H., Hung Y., Lu F., Wu S.-H., Mou C.-Y., Yao M., Chen Y.-C. (2008). Internalization of mesoporous silica nanoparticles induces transient but not sufficient osteogenic signals in human mesenchymal stem cells. Toxicol. Appl. Pharm..

[112] Mahmoudi M., Hofmann H., Rothen-Rutishauser B., Petri-Fink A. (2011). Assessing the in vitro and in vivo toxicity of superparamagnetic iron oxide nanoparticles. Chem. Rev..

[113] Yang C.-Y., Hsiao J.-K., Tai M.-F., Chen S.-T., Cheng H.-Y., Wang J.-L., Liu H.-M. (2011). Direct labeling of hmsc with spio: The long-term influence on toxicity, chondrogenic differentiation capacity, and intracellular distribution. Mol. Imaging Biol..

[114] Soenen S.J., De Cuyper M. (2011). How to assess cytotoxicity of (iron oxide-based) nanoparticles. A technical note using cationic magnetoliposomes. Contrast Media Mol. Imaging.

[115] Schrand A.M., Dai L., Schlager J.J., Hussain S.M. (2012). Toxicity testing of nanomaterials. New Technologies for Toxicity Testing.

[116] Villanueva A., Canete M., Roca A.G., Calero M., Veintemillas-Verdaguer S., Serna C.J., del Puerto Morales M., Miranda R. (2009). The influence of surface functionalization on the enhanced internalization of magnetic nanoparticles in cancer cells. Nanotechnology.

[117] Soenen S.J., Illyes E., Vercauteren D., Braeckmans K., Majer Z., De Smedt S.C., De Cuyper M. (2009). The role of nanoparticle concentration-dependent induction of cellular stress in the internalization of non-toxic cationic magnetoliposomes. Biomaterials.

[118] Naqvi S., Samim M., Abdin M., Ahmed F.J., Maitra A., Prashant C., Dinda A.K. (2010). Concentration-dependent toxicity of iron oxide nanoparticles mediated by increased oxidative stress. Int. J. Nanomed..

[119] Laurent S., Burtea C., Thirifays C., Häfeli U.O., Mahmoudi M. (2012). Crucial ignored parameters on nanotoxicology: The importance of toxicity assay modifications and “cell vision”. PLoS ONE.

[120] Jiang W., Kim B.Y., Rutka J.T., Chan W.C. (2008). Nanoparticle-mediated cellular response is size-dependent. Nat. Nanotechnol..

[121] Kunzmann A., Andersson B., Vogt C., Feliu N., Ye F., Gabrielsson S., Toprak M.S., Buerki-Thurnherr T., Laurent S., Vahter M. (2011). Efficient internalization of silica-coated iron oxide nanoparticles of different sizes by primary human macrophages and dendritic cells. Toxicol. Appl. Pharm..

[122] Mahmoudi M., Simchi A., Milani A., Stroeve P. (2009). Cell toxicity of superparamagnetic iron oxide nanoparticles. J. Coll. Interface Sci..

[123] Liu G., Gao J., Ai H., Chen X. (2013). Applications and potential toxicity of magnetic iron oxide nanoparticles. Small.

[124] Lewinski N., Colvin V., Drezek R. (2008). Cytotoxicity of nanoparticles. Small.

[125] Fischer H.C., Chan W.C. (2007). Nanotoxicity: The growing need for in vivo study. Curr. Opin. Biotechnol..

[126] Monteiro-Riviere N., Inman A., Zhang L. (2009). Limitations and relative utility of screening assays to assess engineered nanoparticle toxicity in a human cell line. Toxicol. Appl. Pharm..

[127] Schneider C.A., Rasband W.S., Eliceiri K.W. (2012). Nih image to imagej: 25 years of image analysis. Nat. Methods.

[128] Kedziorek D.A., Muja N., Walczak P., Ruiz-Cabello J., Gilad A.A., Jie C.C., Bulte J.W. (2010). Gene expression profiling reveals early cellular responses to intracellular magnetic labeling with superparamagnetic iron oxide nanoparticles. Magn. Reson. Med. Off. J. Int. Soc. Magn. Reson. Med..

[129] Buyukhatipoglu K., Clyne A.M. (2011). Superparamagnetic iron oxide nanoparticles change endothelial cell morphology and mechanics via reactive oxygen species formation. J. Biomed. Mater. Res. Part A.

[130] Hsu J.-L., Huang S.-Y., Chow N.-H., Chen S.-H. (2003). Stable-isotope dimethyl labeling for quantitative proteomics. Anal. Chem..

[131] Lin Y.-R., Kuo C.-J., Lin H.Y.-H., Wu C.-J., Liang S.-S. (2014). A proteomics analysis to evaluate cytotoxicity in nrk-52e cells caused by unmodified nano-fe3o4. Sci. World J..

[132] Wilhelm C., Fortin J.-P., Gazeau F. (2007). Tumour cell toxicity of intracellular hyperthermia mediated by magnetic nanoparticles. J. Nanosci. Nanotechnol..

[133] Labusca L., Herea D.-D., Danceanu C.-M., Minuti A.E., Stavila C., Grigoras M., Gherca D., Stoian G., Ababei G., Chiriac H. (2020). The effect of magnetic field exposure on differentiation of magnetite nanoparticle-loaded adipose-derived stem cells. Mater. Sci. Eng. C.

[134] Häfeli U.O., Riffle J.S., Harris-Shekhawat L., Carmichael-Baranauskas A., Mark F., Dailey J.P., Bardenstein D. (2009). Cell uptake and in vitro toxicity of magnetic nanoparticles suitable for drug delivery. Mol. Pharm..

[135] Cengelli F., Maysinger D., Tschudi-Monnet F., Montet X., Corot C., Petri-Fink A., Hofmann H., Juillerat-Jeanneret L. (2006). Interaction of functionalized superparamagnetic iron oxide nanoparticles with brain structures. J. Pharm. Exp. Ther..

[136] Müller K., Skepper J.N., Posfai M., Trivedi R., Howarth S., Corot C., Lancelot E., Thompson P.W., Brown A.P., Gillard J.H. (2007). Effect of ultrasmall superparamagnetic iron oxide nanoparticles (ferumoxtran-10) on human monocyte-macrophages in vitro. Biomaterials.

[137] Mejías R., Gutiérrez L., Salas G., Pérez-Yagüe S., Zotes T.M., Lázaro F.J., Morales M.P., Barber D.F. (2013). Long term biotransformation and toxicity of dimercaptosuccinic acid-coated magnetic nanoparticles support their use in biomedical applications. J. Control. Release.

[138] Shen C.-C., Wang C.-C., Liao M.-H., Jan T.-R. (2011). A single exposure to iron oxide nanoparticles attenuates antigen-specific antibody production and t-cell reactivity in ovalbumin-sensitized balb/c mice. Int. J. Nanomed..

[139] Schlachter E.K., Widmer H.R., Bregy A., Lönnfors-Weitzel T., Vajtai I., Corazza N., Bernau V.J., Weitzel T., Mordasini P., Slotboom J. (2011). Metabolic pathway and distribution of superparamagnetic iron oxide nanoparticles: In vivo study. Int. J. Nanomed..

[140] Malindretos P., Sarafidis P.A., Rudenco I., Raptis V., Makedou K., Makedou A., Grekas D.M. (2007). Slow intravenous iron administration does not aggravate oxidative stress and inflammatory biomarkers during hemodialysis: A comparative study between iron sucrose and iron dextran. Am. J. Nephrol..

[141] Hu J., Wang D., Wang J., Wang J. (2012). Bioaccumulation of fe2o3 (magnetic) nanoparticles in ceriodaphnia dubia. Environ. Pollut..

[142] Taze C., Panetas I., Kalogiannis S., Feidantsis K., Gallios G.P., Kastrinaki G., Konstandopoulos A.G., Václavíková M., Ivanicova L., Kaloyianni M. (2016). Toxicity assessment and comparison between two types of iron oxide nanoparticles in mytilus galloprovincialis. Aquat. Toxicol..

[143] Zhang W., Rittmann B., Chen Y. (2011). Size effects on adsorption of hematite nanoparticles on E. coli cells. Environ. Sci. Technol..

[144] Cao J., Feng Y., Lin X., Wang J., Xie X. (2017). Iron oxide magnetic nanoparticles deteriorate the mutual interaction between arbuscular mycorrhizal fungi and plant. J. Soils Sediments.

[145] Baumann J., Köser J., Arndt D., Filser J. (2014). The coating makes the difference: Acute effects of iron oxide nanoparticles on daphnia magna. Sci. Total Environ..

[146] Zhang Y.-Q., Dringen R., Petters C., Rastedt W., Köser J., Filser J., Stolte S. (2016). Toxicity of dimercaptosuccinate-coated and un-functionalized magnetic iron oxide nanoparticles towards aquatic organisms. Environ. Sci. Nano.

[147] Pappus S.A., Mishra M. (2018). A drosophila model to decipher the toxicity of nanoparticles taken through oral routes. Cellular and Molecular Toxicology of Nanoparticles.

[148] Sayadi M.H., Mansouri B., Shahri E., Tyler C.R., Shekari H., Kharkan J. (2020). Exposure effects of iron oxide nanoparticles and iron salts in blackfish (capoeta fusca): Acute toxicity, bioaccumulation, depuration, and tissue histopathology. Chemosphere.

[149] Hafiz S.M., Kulkarni S.S., Thakur M.K. (2018). In-vivo toxicity assessment of biologically synthesized iron oxide nanoparticles in zebrafish (danio rerio). Biosci. Biotechnol. Res. Asia.

[150] Patel S., Jana S., Chetty R., Thakore S., Singh M., Devkar R. (2019). Toxicity evaluation of magnetic iron oxide nanoparticles reveals neuronal loss in chicken embryo. Drug Chem. Toxicol..

[151] Marin-Barba M., Gavilán H., Gutierrez L., Lozano-Velasco E., Rodríguez-Ramiro I., Wheeler G., Morris C.J., Morales M., Ruiz A. (2018). Unravelling the mechanisms that determine the uptake and metabolism of magnetic single and multicore nanoparticles in a xenopus laevis model. Nanoscale.

[152] Rivero M., Marín-Barba M., Gutiérrez L., Lozano-Velasco E., Wheeler G., Sánchez-Marcos J., Muñoz-Bonilla A., Morris C., Ruiz A. (2019). Toxicity and biodegradation of zinc ferrite nanoparticles in xenopus laevis. J. Nanopart. Res..

[153] Fahmy H.M., Aly E.M., Mohamed F.F., Noor N.A., Elsayed A.A. (2019). Neurotoxicity of green-synthesized magnetic iron oxide nanoparticles in different brain areas of wistar rats. Neurotoxicology.

[154] Volkovova K., Handy R.D., Staruchova M., Tulinska J., Kebis A., Pribojova J., Ulicna O., Kucharská J., Dusinska M. (2015). Health effects of selected nanoparticles in vivo: Liver function and hepatotoxicity following intravenous injection of titanium dioxide and na-oleate-coated iron oxide nanoparticles in rodents. Nanotoxicology.

[155] Awaad A., Seleem A. (2016). Histochemical changes in neonatal liver caused by vaginal instillation of magnetic nanoparticles in pregnant mice. Biotech. Histochem..

